# Analysis of Chemical Heterogeneity in Electrospun Fibers Through Hyperspectral Raman Imaging Using Open-Source Software

**DOI:** 10.3390/polym17131883

**Published:** 2025-07-06

**Authors:** Omar E. Uribe-Juárez, Luis A. Silva Valdéz, Flor Ivon Vivar Velázquez, Fidel Montoya-Molina, José A. Moreno-Razo, María G. Flores-Sánchez, Juan Morales-Corona, Roberto Olayo-González

**Affiliations:** 1 Department of Physics, Metropolitan Autonomous University, Mexico City 09340, Mexico; ouribe@xanum.uam.mx (O.E.U.-J.); mmf99@xanum.uam.mx (F.M.-M.); jamr@xanum.uam.mx (J.A.M.-R.); jmor@xanum.uam.mx (J.M.-C.); 2 Research Vice-Rectory, Faculty of Engineering, University La Salle México, Mexico City 06140, Mexico; guadalupe.flores@lasalle.mx

**Keywords:** electrospun materials, Raman hyperspectral imaging, chemical heterogeneity, spectral unmixing, N-FINDR algorithm, open-source data processing, nanofiber characterization, multicomponent fiber analysis

## Abstract

Electrospinning is a versatile technique for producing porous nanofibers with a high specific surface area, making them ideal for several tissue engineering applications. Although Raman spectroscopy has been widely employed to characterize electrospun materials, but most studies report bulk-averaged properties without addressing the spatial heterogeneity of their chemical composition. Raman imaging has emerged as a promising tool to overcome this limitation; however, challenges remain, including limited sensitivity for detecting minor components, reliance on distinctive high-intensity bands, and the frequent use of commercial software. In this study, we present a methodology based on Raman hyperspectral image processing using open-source software (Python), capable of identifying components present at concentrations as low as 2% and 5%, even in the absence of exclusive bands of high or medium intensity, respectively. The proposed approach integrates spectral segmentation, end member extraction via the N-FINDR algorithm, and analysis of average spectra to map and characterize the chemical heterogeneity within electrospun fibers. Finally, its performance is compared with the traditional approach based on band intensities, highlighting improvements in sensitivity and the detection of weak signals.

## 1. Introduction

Electrospinning is a simple and affordable method for producing nanofibers, offering a large specific surface area and highly porous structures, with diameters ranging from the nanometric to the micrometric scale. These electrospun fibers enable the combination of multiple polymers, drugs, and biomolecules, providing synergistic properties ideal for applications in areas such as tissue engineering, biomedicine, and controlled drug release [1,2,3].

Raman spectroscopy has been widely used to study the physicochemical characteristics of electrospun materials [4,5,6,7,8,9,10,11,12,13]. However, in most of the studies reported in the literature that employ Raman spectroscopy for the characterization of composite electrospun fibers, the analysis is limited to “bulk” properties, that is, the spectral signature is recorded at a few points or specific zones of the material, under the assumption of structural homogeneity across the fiber [14,15]. Since one of the main advantages of electrospinning is precisely the ability to combine multiple compounds into a single structure, there has been a growing interest in developing techniques that allow the study of the chemical and spatial heterogeneity within the fibers.

Although a few studies have reported the use of Raman imaging to determine the spatial distribution of components within electrospun fibers, these typically rely on systems where the components exhibit strong and exclusive Raman bands, and where the minority compound is present at concentrations of 10% or higher (relative to the total mass) [15,16,17,18,19], and hyperspectral data processing is usually performed using commercial software, which reduces accessibility, reproducibility, and flexibility in the analysis [15,17,18,19,20]. These conditions highlight the challenges and limitations associated with analyzing the complete chemical structure of heterogeneous electrospun fibers.

In this work, we propose a methodology for analyzing the chemical heterogeneity of composite electrospun fibers using hyperspectral Raman imaging. The proposed method does not rely on the presence of strong exclusive Raman bands between components and is capable of detecting minority compounds present at concentrations as low as 5% or 2% of the total mass. Furthermore, the methodology is implemented using open-source software, ensuring accessibility and adaptability for other researchers and allowing customization according to specific needs.

The proposed approach consists of image and spectral segmentation to identify the pixels and bands with the most relevant information about the compounds, followed by the application of an algorithm to determine the end members (N-FINDR) [21], and their relationship with the present compounds. From the end member projections, abundance maps are generated, the spatial distribution of the fiber components is segmented, and average spectra are obtained, enabling a detailed assessment of chemical heterogeneity within the fibers.

To validate the method, three polymeric blend systems were analyzed.

PLA/HA (64.3% polylactic acid, 35.7% hydroxyapatite): This system has been studied for bone tissue regeneration applications [22] and features strong and exclusive Raman bands for each component. Thus, mapping its chemical heterogeneity using hyperspectral Raman imaging is expected to be relatively straightforward.

PCL/Collagen (98% polycaprolactone, 2% collagen): This system has been investigated in vitro with fibroblasts as a potential biomaterial [23]. While it presents exclusive bands of medium intensity, its low collagen content makes their chemical heterogeneity analysis moderately challenging.

PLA/PVA (95% polylactic acid, 5% polyvinyl alcohol): This system was selected because PVA is water-soluble [24], enabling validation of the component distribution through scanning electron microscopy (SEM) after fiber washing [25]. However, its components exhibit exclusive Raman bands of low intensity, making the chemical heterogeneity analysis particularly difficult.

Finally, the performance of the proposed method is compared to the traditional approach based on exclusive band intensities, evaluating its sensitivity to detect minority components and its ability to identify weak bands that could go unnoticed in conventional analyses.

## 2. Experimental Section

### 2.1. Materials

Polylactic acid (PLA), polycaprolactone (PCL), collagen, polyvinyl alcohol (PVA), hydroxyapatite (HA), chloroform, methanol, ethanol, acetic acid, and deionized water were used for the fabrication of electrospun fibers. All reagents, except deionized water, were purchased from Sigma Aldrich (distributed by MilliporeSigma, Mexico City, Mexico). 

### 2.2. Electrospinning

For the PLA/HA electrospun fibers (64.3% PLA and 35.7% HA), 3.6 g of PLA was dissolved in a chloroform and ethanol mixture (9:1, *v*/*v*) under stirring for 3 h at room temperature. Subsequently, 2 g of HA was added, and the solution was stirred for an additional 2 h. The resulting mixture was loaded into a 20 mL syringe with an 18-gauge needle and electrospun at 25 kV, with a needle-to-collector distance of 15 cm and a flow rate of 5 mL/h.

For the PCL/collagen electrospun fibers, the solutions for electrospinning were prepared, separately dissolving PCL in a 90/10 (*v*/*v*) chloroform/methanol mixture (150 mg/mL), and stirring for 5 h at room temperature, and dissolving collagen in acetic acid (20 mg/mL) under constant stirring for 24 h at room temperature. Both solutions were then mixed and stirred to obtain a final mixture containing 98% polycaprolactone (PCL) and 2% collagen by mass. The final solution was loaded into a 20 mL syringe equipped with a 22-gauge needle and electrospun at 15 kV, with a needle-to-collector distance of 15 cm and a flow rate of 5 mL/h.

For the PLA/PVA electrospun fibers, the solutions for electrospinning were prepared by separately dissolving PLA in chloroform (178 mg/mL), under stirring for 3 h at room temperature, and dissolving PVA in deionized water (200 mg/mL) under constant stirring for 3 h at room temperature. Both solutions were then mixed and stirred at 40 °C for 24 h to obtain a final mixture containing 95% PLA and 5% PVA by mass. The resulting solution was loaded into a 20 mL syringe equipped with a 19-gauge needle and electrospun at 25 kV, with a needle-to-collector distance of 19 cm and a flow rate of 3 mL/h.

### 2.3. Raman Imaging

Raman images were acquired using a WITec Alpha 300 RA+ confocal micro-Raman spectrometer (Oxford Instruments, Ulm, Germany) equipped with a 532 nm laser at 75 mW power. For the PLA/HA electrospun fibers, a Zeiss EC Epiplan 20× objective (NA 0.4) (Zeiss, Jena, Germany) was used, capturing an image area of 174 × 154 µm with 100 lines per image, 100 points per line, and an integration time of 5 s. For the PCL/collagen fibers, a Zeiss EC Epiplan 50× objective (NA 0.75) was employed, with an image area of 80 × 80 µm, 100 lines per image, 100 points per line, and an integration time of 4 s, and the image spatial resolution was Δx=Δy=811 nm and Δz=3 μm [26]. For the PLA/PVA fibers, the same 50× objective (NA 0.75) was used, imaging an area of 95 × 95 µm, with 100 lines per image, 100 points per line, and an integration time of 5 s, and the image spatial resolution was Δx=Δy=433 nm and Δz=842 nm [26].

### 2.4. Raman Spectra

Raman spectra of PLA, HA, PCL, collagen, and PVA were collected using an integration time of 4 s and 20 accumulations.

### 2.5. Data Processing

Raman spectra and images were processed using Python (version: 3.12.4) with the RamanSPy (version: 0.2.10), NumPy (version: 1.26.4), Matplotlib (version: 1.26.4), OpenCV (cv2, version: 4.11.0), and SciPy (version: 1.12.0) libraries. Spectral preprocessing included cosmic ray removal using the Whitaker–Hayes method, spectral smoothing with the Savitzky–Golay filter, and baseline correction using the Doubly Reweighted Penalized Least Squares (DRPLS) method.

### 2.6. Spectral Segmentation with the N-FINDER Algorithm

To identify pixels containing compound information in the Raman images, baseline correction of the spectra was first performed using the DRPLS method [20]. Abundance maps for each compound were generated based on the wavenumber of the most intense Raman band [27]. These maps were binarized using a threshold to exclude pixels without relevant information [28]. Morphological erosion and dilation operations with a 1 × 1 kernel were then applied to remove pixels with anomalous intensities caused by cosmic rays [29]. The resulting images were summed and binarized again.

For Raman band segmentation, regions of interest were cropped and processed as described above. The processed bands were incorporated into synthetic spectra constructed with the original spectral axis and assigned an intensity value of 1 [20]. The N-FINDER algorithm was used to obtain the end members [21].

### 2.7. Abundance Maps

Abundance maps were generated using the exclusive band intensities of each compound and the projections of the end members obtained with the N-FINDER algorithm.

### 2.8. Average Spectra

Average spectra were calculated using the abundance maps of the end members. These maps were binarized with a threshold to select only pixels exhibiting the highest signal intensity for the corresponding end member. The intersection between maps was computed to remove pixels with strong signals from multiple compounds. The resulting maps were used as masks to select pixels for averaging.

### 2.9. Statistical Analysis

The Shapiro–Wilk [30] and Levene tests [31] were performed to assess the normality and homogeneity of variances of the data, respectively. Since the data did not meet the assumptions of a normal distribution and equal variances, the non-parametric Mann–Whitney U test [32] was used to evaluate whether statistically significant differences existed between the two groups.

### 2.10. Signal-to-Noise Ratio

The signal-to-noise ratio was calculated using the following expression, similar to that reported in [33]:

$$SNR=\frac{S}{\sigma_{y}}$$

where S is the height of the most intense Raman band measured from the baseline, and $\sigma_{y}$ is the standard deviation of the S heights relative to the nearest neighboring pixels.

**Figure 1:**
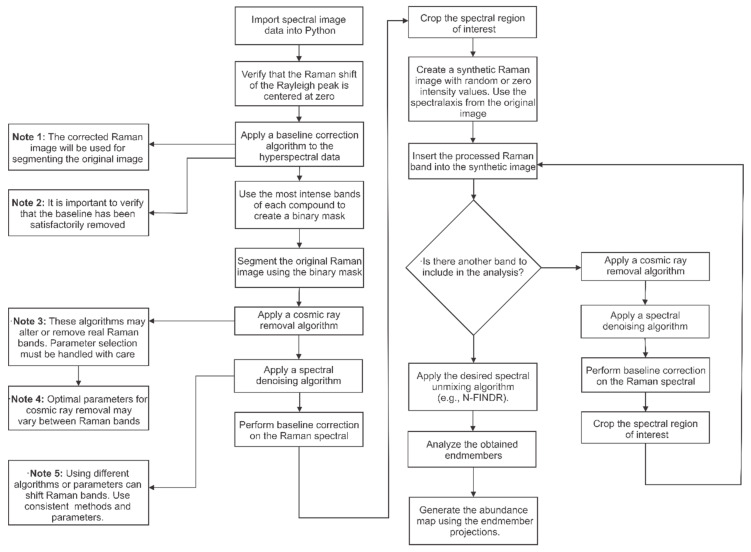
Detailed step-by-step procedure to reproduce the methodology proposed in this work, along with some notes and recommendations for best practices [34].

Flowchart: Raman Hyperspectral Data Processing (Figure 1).

### 2.11. Computational Resources

Data processing was performed using a computer equipped with an AMD Ryzen 5 7535HS processor (AMD, Santa Clara, CA, USA) with Radeon Graphics at 3.30 GHz, 16 GB of RAM, and an NVIDIA GeForce RTX 4050 graphics card. The average computation time for each analyzed system was 147 s.

## 3. Results and Discussion

### 3.1. PLA/HA Electrospun Fiber

**Figure 2:**
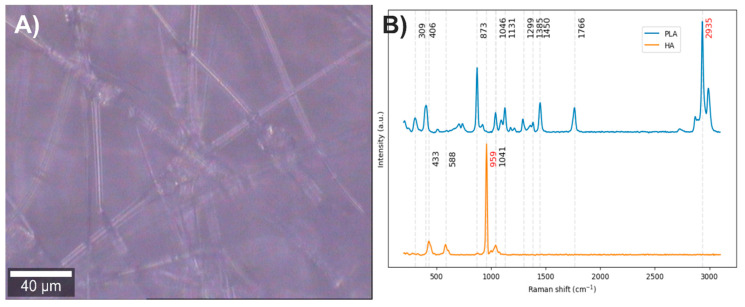
(**A**) PLA/HA electrospun fiber image (20×). (**B**) PLA and HA Raman spectra. The red numbers indicate the wavenumbers of exclusive bands corresponding to each component.

Electrospun fibers composed of PLA with 35.7% HA were obtained (Figure 2A). The vibrational modes associated with the chemical structure of each component were studied by Raman spectroscopy (Figure 2B). In the Raman spectrum of PLA, the bands centered at 2935 cm^−1^ correspond to C–H stretching vibrations: the band at 2871 cm^−1^ is attributed to stretching, while those at 2935 cm^−1^ and 2991 cm^−1^ correspond to symmetric and asymmetric stretching, respectively. The band at 1766 cm^−1^ is related to the carbonyl group (C=O) stretching. Bands between 1137 and 1450 cm^−1^ are assigned to C–H bending modes; however, the interpretation of these bands varies among authors, reflecting a lack of clear consensus. Most agree that bands in this region correspond to in-plane and out-of-plane bending, although discrepancies remain regarding the exact symmetry of these vibrational modes [35,36,37,38,39,40]. Considering that in alkanes, the antisymmetric and symmetric bending of methyl groups occur between 1470 and 1430 cm^−1^ and between 1395 and 1365 cm^−1^, respectively, and that the antisymmetric band near 1460 cm^−1^ has medium intensity while the symmetric band is usually weak in the absence of conjugation enhancing Raman activity [41], it is likely that the bands at 1450 and 1385 cm^−1^ correspond to antisymmetric and symmetric in-plane bending, respectively. The bands at 1046 and 873 cm^−1^ are attributed to C–CH_3_ and C–O–C stretching vibrations, respectively, while the bands at 406 and 309 cm^−1^ correspond to molecular skeleton deformations involving C–CH_3_, C–C–O, and C–O–C bonds [35,36,37,38,39,40].

In the Raman spectrum of hydroxyapatite, the bands at 1041 and 959 cm^−1^ are assigned to asymmetric and symmetric stretching of P–O bonds, respectively, while the bands at 588 and 433 cm^−1^ correspond to bending modes of these same bonds [42,43].

**Figure 3:**
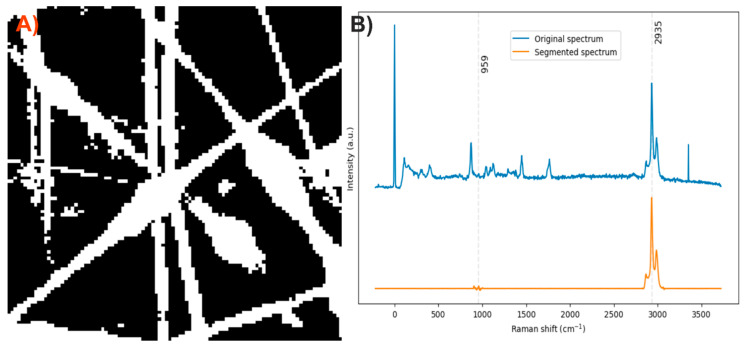
(**A**) Segmented image of the PLA/HA electrospun fiber identifying pixels with relevant information. (**B**) Raman spectra before and after band segmentation.

After analyzing the components of the PLA/HA electrospun fiber, it was identified that the bands at 2935 and 959 cm^−1^ can be used to distinguish the presence of PLA and HA, respectively, through a comparison of exclusive band intensities and by the image and Raman spectra segmentation method proposed in this work. The acquired Raman image and its spectra were segmented using the C–H stretching band and the symmetric P–O stretching band. Approximately 32% of the pixels in the Raman image contained relevant information about the scaffold components (Figure 3).

**Figure 4:**
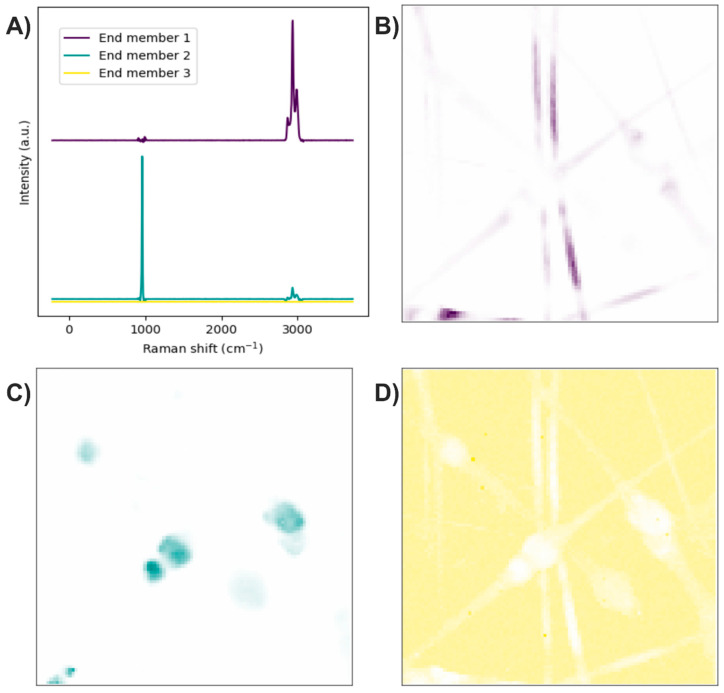
PLA/HA electrospun fibers. (**A**) End members calculated with the N-FINDR algorithm. (**B**), (C), and (**D**) Abundance maps of end members 1, 2, and 3, respectively.

The end members of the Raman image were determined using the N-FINDR algorithm (Figure 4A). The Raman bands of the first end member are related to C–H stretching, thus representing the presence of PLA (Figure 4B). The second end member mainly exhibits bands associated with symmetric P–O stretching, although a weak band corresponding to C–H stretching is also observed, indicating the presence of HA accompanied by PLA at a lower concentration (Figure 4C). The third end member lacks Raman bands, suggesting it is related to noise in the Raman image (Figure 4D).

**Figure 5:**
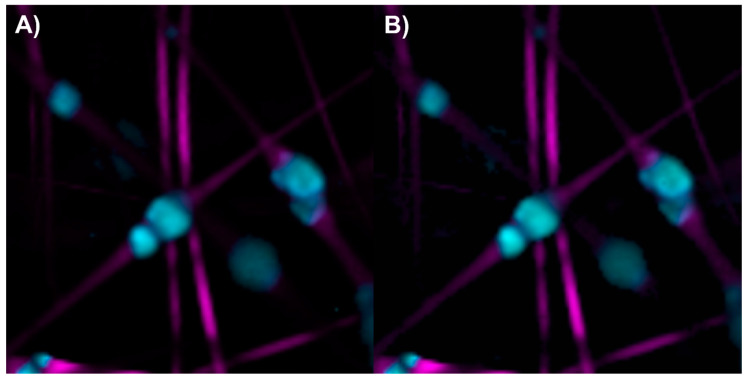
Abundance maps of PLA (magenta) and HA (cyan) obtained by (**A**) the characteristic band of each compound and (**B**) image and Raman spectra segmentation combined with the N-FINDR algorithm.

The abundance map of the PLA/HA electrospun components was constructed using two different methods: by combining intensity maps of the bands at 959 and 2935 cm^−1^ (Figure 5A) and by applying the image and Raman spectra segmentation method combined with the N-FINDR algorithm (Figure 5B). No significant differences were observed between the two methods for this scaffold. HA is concentrated, forming beads on the PLA fibers.

**Figure 6:**
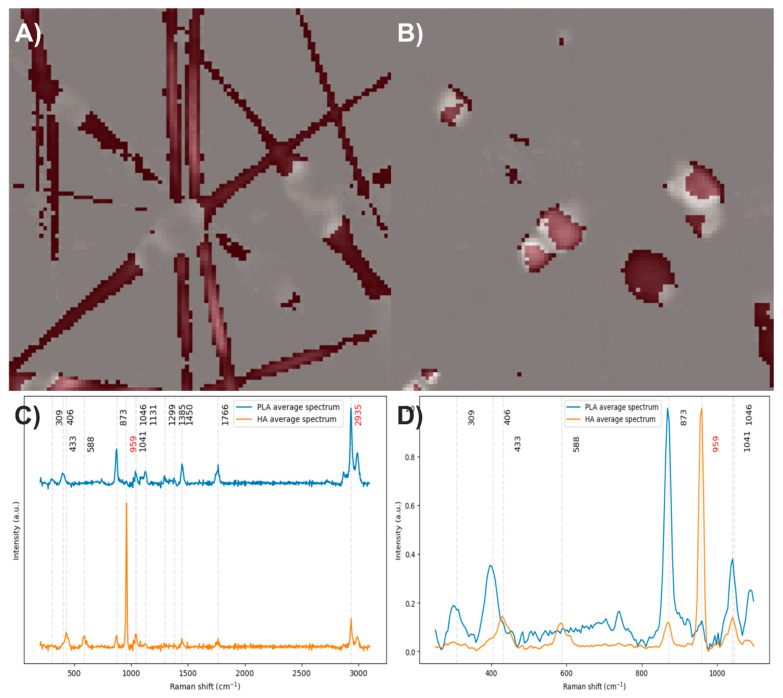
(**A**) and (**B**) Pixels of the image used to calculate the average spectra of PLA and HA, respectively. (**C**) Average spectra of PLA and HA. (**D**) Baseline-corrected, denoised, and normalized average spectra.

To obtain the average spectrum of PLA, 1746 spectra were averaged (Figure 6A), while 507 spectra were averaged for HA (Figure 6B). When comparing both average spectra (Figure 6C), the HA spectrum shows the characteristic bands of this compound, as expected, but also exhibits bands related to PLA (1450, 1766, and 2935 cm^−1^), which agrees with the end member analysis and confirms that HA is not isolated within the fibers but accompanied by PLA at a lower concentration. After baseline correction, denoising, normalization, and comparison of the average spectra, a barely perceptible band at 959 cm^−1^ is observed in the PLA average spectrum (Figure 6D), suggesting the presence of HA in the PLA fibers, albeit at very low concentrations not detectable by the abundance maps.

### 3.2. PCL/Collagen Electrospun Fiber

**Figure 7:**
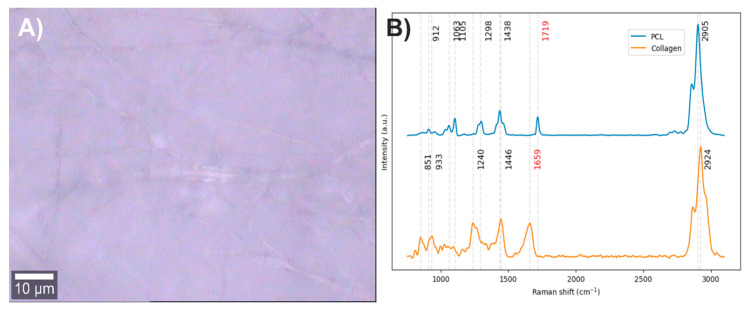
(**A**) PCL/Collagen electrospun fiber image (50×). (**B**) PCL and collagen Raman spectra. The red numbers indicate the wavenumbers of exclusive bands corresponding to each component.

Electrospun fibers composed of PCL with 2% collagen were obtained (Figure 7A). The vibrational modes related to the chemical structure of each component were studied through Raman spectroscopy (Figure 7B). In the Raman spectrum of PCL, the bands centered at 2905 cm^−1^ are related to the C-H stretching vibrations, the band at 1719 cm^−1^ to the carbonyl group (C=O) stretching, the bands at 1438 and 1298 cm^−1^ to in-plane and out-of-plane C-H bending modes, and the bands between 850 and 1010 cm^−1^ to the stretching of the polymer skeleton of C-C, C-O, and C-COO bonds [44,45]. In the Raman spectrum of collagen, the band at 2924 cm^−1^ is related to the C-H stretching vibrations, the band at 1659 cm^−1^ to amide I, the band at 1446 cm^−1^ to in-plane C-H bending modes, the band at 1260 cm^−1^ to amide III, the band at 933 cm^−1^ to the stretching of C-C bonds in the protein skeleton, and the band at 851 cm^−1^ to the stretching of C-C bonds in proline and hydroxyproline rings [46,47].

**Figure 8:**
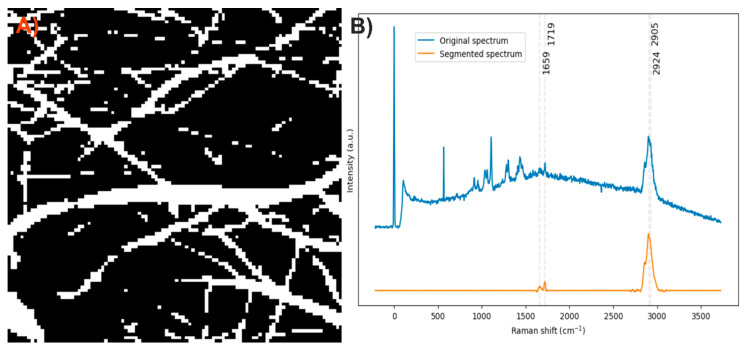
(**A**) Segmented image of the PCL/collagen electrospun fiber identifying pixels with relevant information. (**B**) Raman spectra before and after band segmentation.

After analyzing the components of the PCL/collagen electrospun fiber, it was found that the bands at 1719 and 1659 cm^−1^ can be used to identify the presence of PCL and collagen, respectively, through the method of exclusive band intensities between components. For the image segmentation and Raman spectra method, it was also decided to include the band for the stretching of C-H bonds, as they are the most intense bands in the two components and are phase-shifted. The Raman image acquired was segmented using the band for the stretching of C-H bonds (common to both components). Approximately only 22% of the pixels in the Raman image contained information about the electrospinning components (Figure 8A). The Raman spectra were segmented, leaving only the information from the bands related to amide I, the carbonyl group, and the C-H stretching (Figure 8B).

**Figure 9:**
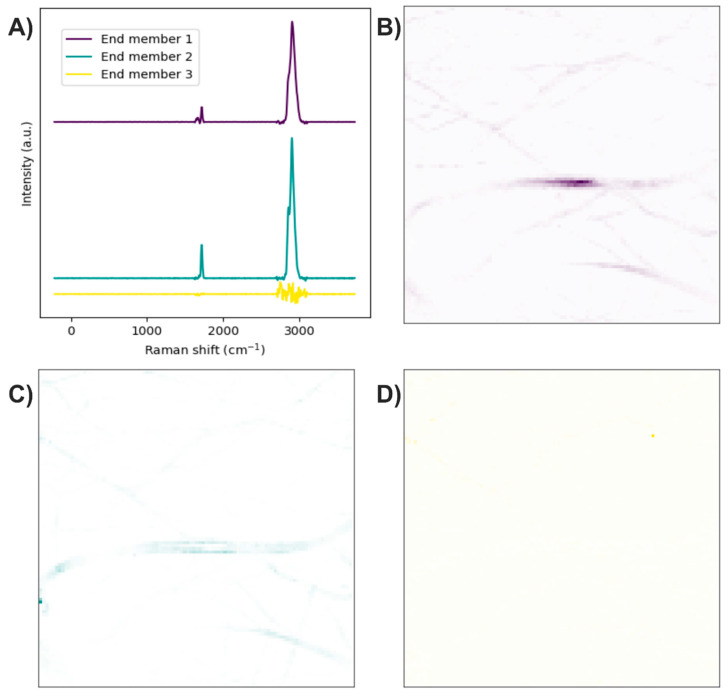
PCL/collagen electrospun fibers. (**A**) End members calculated with the N-FINDR algorithm. (**B**), (**C**), and (**D**) Abundance maps of end members 1, 2, and 3, respectively.

The end members of the Raman image were determined using the N-FINDR algorithm (Figure 9A). The Raman bands of the first end member are related to amide I, the carbonyl group, and C-H stretching; therefore, the first end member indicates the presence of PCL with collagen (Figure 9B). The Raman bands of the second end member are related to the carbonyl group and C-H stretching; thus, the second end member indicates the presence of PCL (Figure 9C). In the third end member, the absence of Raman bands indicates that this end member is associated with the noise in the Raman image (Figure 9D).

**Figure 10:**
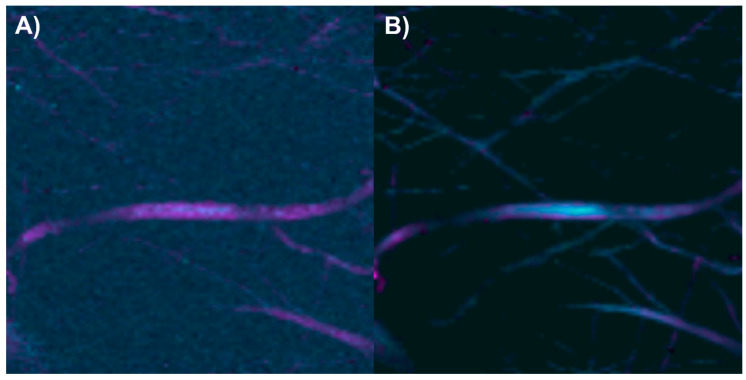
Abundance maps of PCL (magenta) and collagen (cyan) obtained by (**A**) the characteristic band of each compound and (**B**) image and Raman spectra segmentation combined with the N-FINDR algorithm.

The abundance map of the PCL/collagen electrospun components was constructed using two different methods: by combining the intensity maps of the bands at 1659 and 1719 cm^−1^ (Figure 10A) and by applying the image and Raman spectra segmentation method combined with the N-FINDR algorithm (Figure 10B). For this electrospun fiber, it is observed that the method of exclusive band intensities between components has trouble identifying pixels with a lower collagen concentration and distinguishing the noise in the Raman image. In contrast, the image and Raman spectra segmentation method combined with the N-FINDR algorithm seems to have a greater capacity to identify pixels with a lower collagen concentration, as well as to distinguish between the electrospun components and the noise in the Raman image.

**Figure 11:**
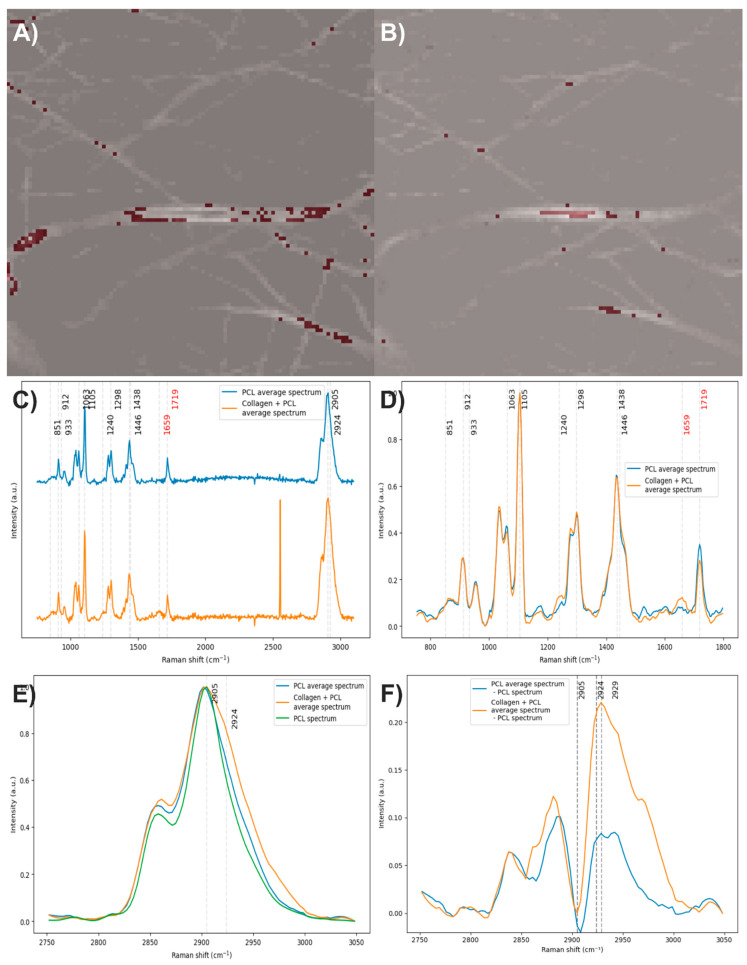
(**A**) and (**B**) Pixels of the image used to calculate the average spectra of PCL and PCL with collagen, respectively. (**C**) Average spectra of PCL and PCL with collagen. (**D**,**E**) Baseline-corrected, denoised, and normalized average spectra. (**F**) Average spectra after subtracting the spectrum of electrospun PCL without collagen. The red numbers indicate the wavenumbers of exclusive bands corresponding to each component.

To obtain the average spectrum of PCL, 159 individual spectra were averaged (Figure 11A), while for the average spectrum of PCL with collagen, 46 spectra were considered (Figure 11B). When comparing both average spectra (Figure 11C), the PCL spectrum displays the characteristic bands of this polymer, as expected. In contrast, the average spectrum of PCL with collagen shows, in addition to the typical PCL bands, the presence of the amide I band, confirming the incorporation of collagen into the fibers (Figure 11D). After baseline correction, denoising, normalization, and comparison of the average spectra, it was observed that the average spectrum of PCL with collagen also exhibits the amide III band, along with a slight decrease in the intensity of the carbonyl group. It is worth noting that the average spectrum of PCL also shows a very faint signal in the region corresponding to the amide I band. When analyzing the C–H stretching region, which corresponds to the most intense bands in both components, and comparing them to the spectrum of electrospun PCL without collagen, an increase in the area under the curve was identified in both average spectra (Figure 11E). This increase is more prominent in the average spectrum of PCL with collagen, suggesting an additional contribution in this region, likely due to collagen. To estimate this possible contribution, the average spectrum of electrospun PCL without collagen was subtracted from the average spectra (Figure 11F). This operation revealed the presence of two bands centered at 2929 cm^−1^, which could correspond to weak signals associated with collagen. These results suggest that the C–H stretching region could be used as a marker to analyze the distribution of components in multicomponent materials, particularly when no strong, exclusive Raman bands are available and one of the compounds is present in concentrations as low as 2%. However, caution is advised, as variations in the C–H stretching region can also arise from other factors, such as focal plane shifts during spectral acquisition. Therefore, whenever possible, it is recommended to confirm the presence of the predicted compound through another distinctive Raman band that can help validate its identification.

### 3.3. PLA/PVA Electrospun Fiber

**Figure 12:**
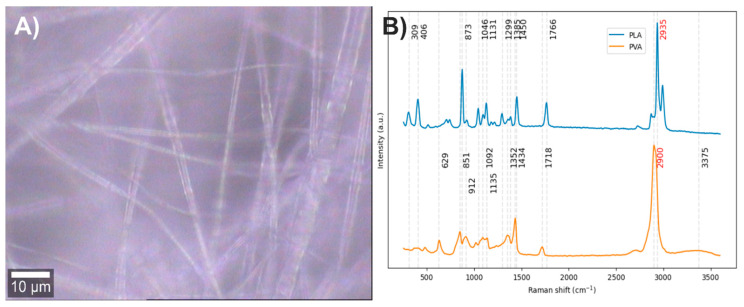
(**A**) PLA/PVA electrospun fiber image (50×). (**B**) PLA and PVA Raman spectra. The red numbers indicate the wavenumbers of the most intense Raman bands corresponding to each component.

Electrospun fibers composed of PLA with 5% PVA were obtained (Figure 12A). The vibrational modes associated with the chemical structure of each component were studied by Raman spectroscopy (Figure 12B). Since the Raman spectrum of PLA has previously been discussed, this section focuses solely on the spectrum corresponding to PVA. In the Raman spectrum of PVA, the band centered at 3378 cm^−1^ is attributed to O–H stretching vibrations, the band at 2900 cm^−1^ corresponds to symmetric stretching of C–H bonds, and the band at 1718 cm^−1^ is related to the stretching of the carbonyl group (C=O) from acetate residues. For the bands at lower frequencies than the carbonyl group, interpretations vary among different authors, reflecting a lack of clear consensus. However, most agree that Raman bands between 1200 and 1500 cm^−1^ are associated with bending modes of C–H and O–H bonds; bands between 1020 and 1150 cm^−1^ correspond to C–O stretching vibrations; bands at 912 and 851 cm^−1^ are attributed to C–C stretching; while the band at 629 cm^−1^ has diverse interpretations [24,48,49,50]. In order to improve the interpretation of the Raman spectrum of PVA, vibrational modes of alkenes and alcohols in the 750 to 1500 cm^−1^ region were reviewed. Nevertheless, no conclusive information was found to improve the spectral interpretation. The available data agree with previous reports, suggesting that the region between 1200 and 1500 cm^−1^ likely corresponds to a mixture of O–H and C–H bending modes with medium to high Raman intensities [41,51].

**Figure 13:**
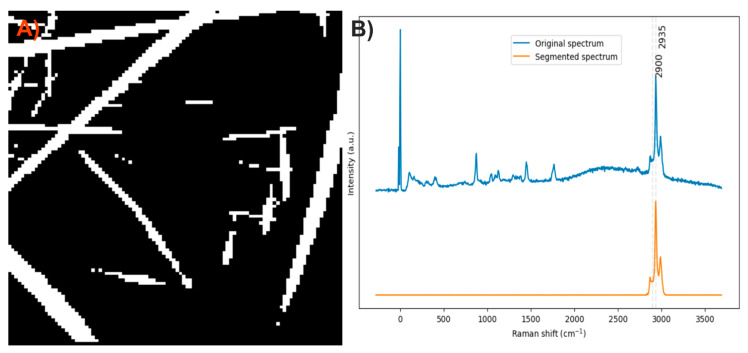
(**A**) Segmented image of the PLA/PVA electrospun fiber identifying pixels with relevant information. (**B**) Raman spectra before and after band segmentation.

After analyzing the components of the PLA/PVA electrospun fiber, it was found that the bands at 406 and 629 cm^−1^ can be used to identify the presence of PLA and PVA, respectively, through the method of exclusive band intensities between components. For the image segmentation and Raman spectra method, the bands corresponding to C-H stretching were selected, as they are the most intense in both compounds, exhibit different profiles, and are also shifted. Approximately only 15% of the pixels in the Raman image contained relevant information about the electrospinning components (Figure 13A). The Raman spectra were segmented, leaving only the information related to the C-H stretching bands (Figure 13B).

**Figure 14:**
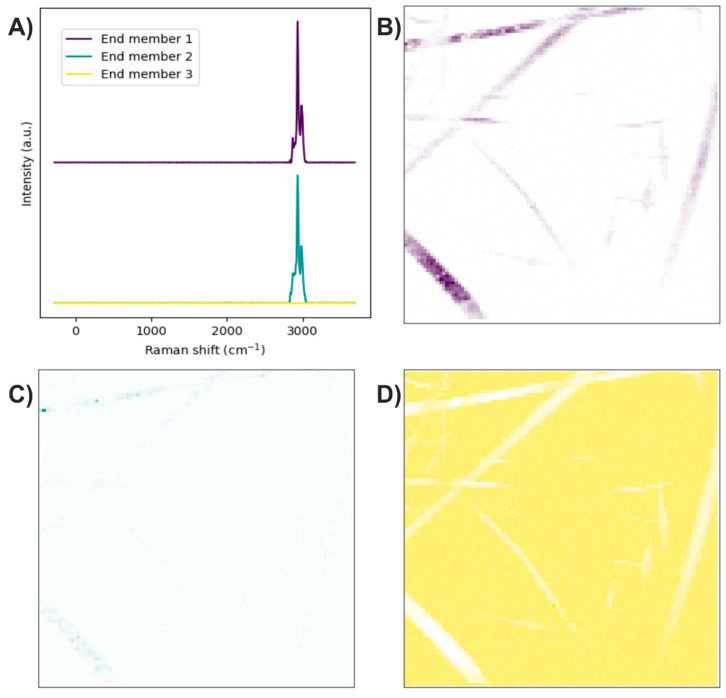
PLA/PVA electrospun fibers. (**A**) End members calculated with the N-FINDR algorithm. (**B**), (**C**), and (**D**) Abundance maps of end members 1, 2, and 3, respectively.

The end members of the Raman image were determined using the N-FINDR algorithm (Figure 14A). The Raman spectrum of the first end member shows a shape similar to the C-H stretching band of PLA, indicating the presence of this compound (Figure 14B). Regarding the second end member, its Raman bands present a profile similar to that of the PLA C-H stretching bands, along with an additional contribution between 2811 and 2932 cm^−1^. Since the symmetric C-H stretching in PVA occurs at 2900 cm^−1^, this second end member could indicate the presence of PLA with PVA (Figure 14C). In the third end member, the absence of Raman bands indicates that this end member is associated with the noise in the Raman image (Figure 14D).

**Figure 15:**
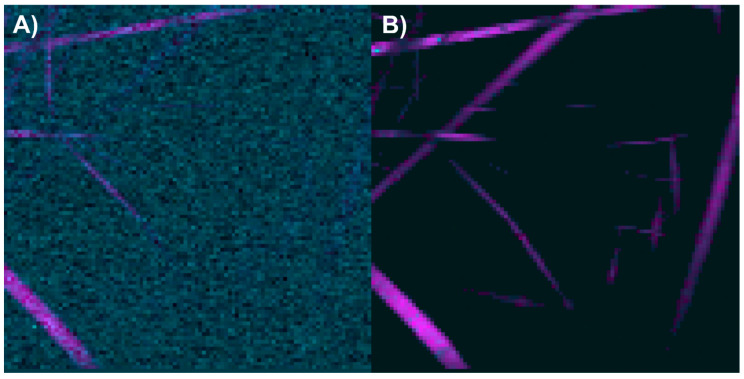
Abundance maps of PLA (magenta) and PVA (cyan) obtained by (**A**) the characteristic band of each compound and (**B**) image and Raman spectra segmentation combined with the N-FINDR algorithm.

The abundance map of the PLA/PVA electrospun components was constructed using two different methods: by combining intensity maps of the bands at 406 and 629 cm^−1^ (Figure 15A), and by applying the image and Raman spectra segmentation method combined with the N-FINDR algorithm (Figure 15B). For this scaffold, it was observed that the method based on exclusive band intensities failed to adequately identify pixels containing PVA, likely due to the weak intensity of the 629 cm^−1^ band, which at a 5% concentration could be indistinguishable from the Raman image noise. In contrast, the image and Raman spectra segmentation method combined with the N-FINDR algorithm demonstrated a greater ability to identify pixels with PVA presence, as well as to distinguish between the electrospun components and the noise in the Raman image.

**Figure 16:**
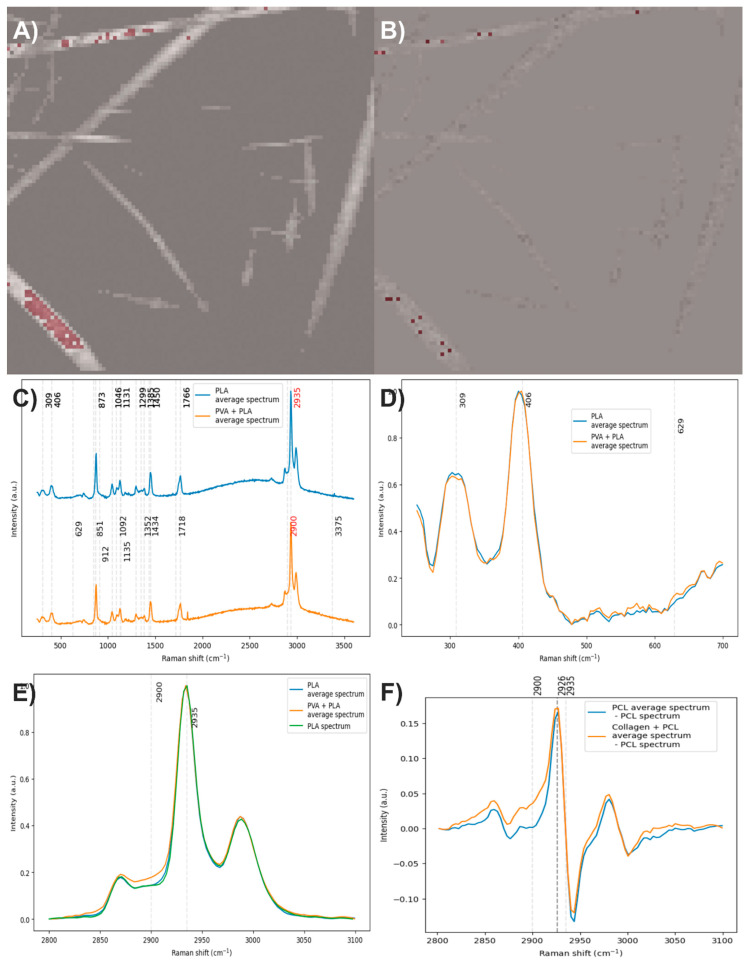
(**A**) and (**B**) Pixels of the image used to calculate the average spectra of PLA and PLA with PVA, respectively. (**C**) Average spectra of PLA and PLA with PVA. (**D**,**E**) Baseline-corrected, denoised, and normalized average spectra. (**F**) Average spectra after subtracting the spectrum of electrospun PLA without PVA. The red numbers indicate the wavenumbers of the most intense Raman bands corresponding to each component.

To obtain the average spectrum of PLA, a total of 97 spectra were averaged (Figure 16A), whereas for the average spectrum of PVA with PLA, only 16 spectra were used to compute the average spectrum (Figure 16B). The average spectrum of PLA exhibits all the characteristic Raman bands of this polymer, as expected. In contrast, the average spectrum of PVA with PVA does not show any distinct bands attributable to PVA (Figure 16C). After baseline correction, denoising, normalization, and comparison of the average spectra, it was observed that the band at 629 cm^−1^ is absent in the average spectrum of PVA with PLA. This result may be attributed to the limited number of spectra used to calculate the average PVA spectrum. When averaged, low-intensity bands may not be sufficiently reinforced and may therefore remain undetected (Figure 16D). When analyzing the C–H stretching region, which corresponds to the most intense bands in both components, and comparing them to the spectrum of electrospun PLA without PVA, an increase in the area under the curve was identified only in PLA with the PVA average spectrum (Figure 16E), likely due to PVA. To estimate this possible contribution, the spectrum of electrospun PLA without PVA was subtracted from the average spectra (Figure 16F). This operation revealed the presence of two bands centered at 2926 cm^−1^, and this wavenumber does not match the typical PVA peak at ~2900 cm^−1^.

The observed shift in the PVA band may be attributed to interfacial interactions between PLA and PVA, since changes in the chemical environment can induce shifts in Raman bands [52]. Alternatively, this shift could be related to the limited number of spectra used to calculate the average PVA spectrum, as previously mentioned.

**Figure 17:**
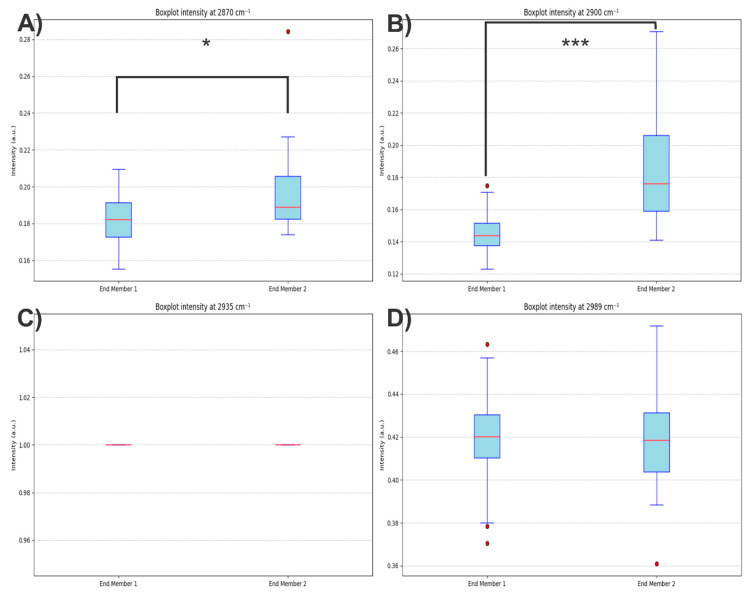
Boxplots for the spectral intensities at (**A**) 2870, (**B**) 2900, (**C**) 2935, and (**D**) 2989 cm^−1^ for two groups of spectra (End member 1 and End member 2). The asterisk symbol (*) in (A) indicates statistical significance at *p* < 0.05, while the triple asterisk (***) in (B) indicates *p* < 0.001. Red dots represent outliers in each distribution.

To determine whether the additional contributions observed in end member 2 were solely attributable to PLA, statistical tests were performed on the spectral intensities at 2870, 2900, 2935, and 2989 cm^−1^. The results revealed significant differences between pixel groups, with higher intensities in end members 1 and 2, confirming that the second end member is not exclusively associated with PLA (Figure 17).

**Figure 18:**
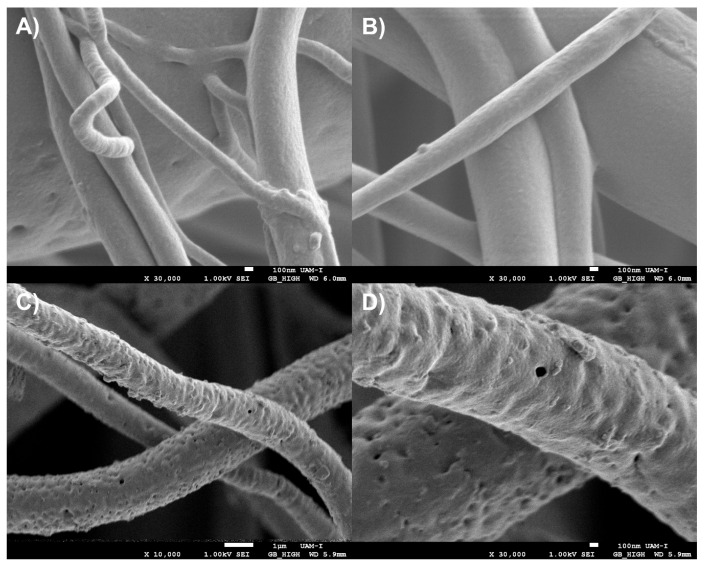
Scanning electron microscopy of PLA/PVA electrospun fibers (**A**,**B**) before and (**C**,**D**) after washing them with water to remove the PVA at different magnifications.

As previously discussed, the use of the most intense Raman bands, especially when they exhibit distinct profiles or are spectrally shifted, appears to be helpful in determining the spatial distribution of electrospun components using the proposed method. Nevertheless, it is crucial to find additional evidence to confirm the identity and distribution of the components. In this case, due to the lack of a confirmatory Raman band for PVA, scanning electron microscopy (SEM) was employed. PVA is a water-soluble polymer [24], and washing PVA-containing materials typically results in porous structures [25]. After washing the PLA/PVA fibers and imaging them by SEM, pores were observed on the surface of the PLA fibers. Since PLA is not water-soluble [53], the presence of pores indicates the original distribution of PVA within the PLA fibers prior to washing (Figure 18). This distribution observed via SEM is consistent with the component distribution inferred using the hyperspectral method proposed in this work.

### 3.4. Overall Intensity and Signal-to-Noise Ratio (SNR)

The ability to extract analytical information from any spectroscopic technique is generally limited by the signal-to-noise ratio (SNR) [33]. Quantitative analysis is not feasible when the SNR falls below two [54].

In our study, the system with the lowest SNR was PLA/HA, likely due to the image being acquired with an objective lens of lower numerical aperture (NA). Nevertheless, the chemical heterogeneity of this system was relatively straightforward to analyze, even using the traditional approach based on exclusive band intensities, which is consistent given that both compounds exhibit intense and exclusive Raman bands, as shown in the intensity map. Although the SNR at some points along the fibers in this system approaches two, the analysis proposed thus far is not quantitative in nature.

The PLA/collagen and PLA/PVA systems exhibited SNR values above two, confirming the reliability of our results [54].

**Figure 19:**
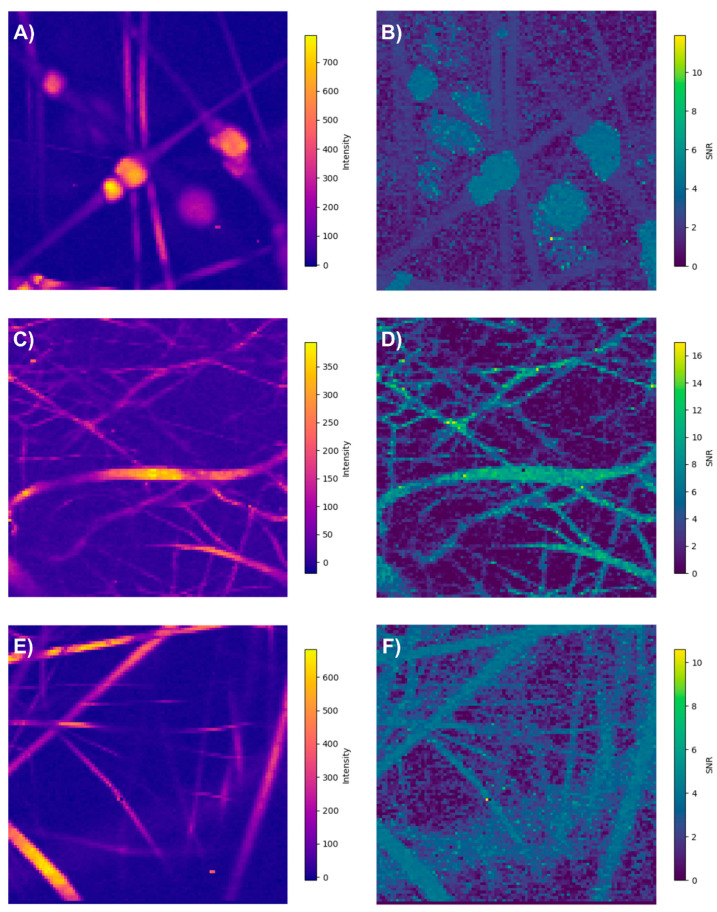
Recorded spectra overall intensity and signal-to-noise ratio (SNR): (**A**,**B**) PLA/HA, (**C**,**D**) PCL/Callagen, and (**E**,**F**) PLA/PVA.

An interesting finding is that, when employing the spatial SNR, pixels with low intensity but a high SNR can be identified, containing relevant information about the analyzed sample (Figure 19). This underscores the importance of developing spectral processing techniques that enable the exploitation of such spectral information.

## 4. Conclusions

In this work, a method based on open-source software (Python) was developed to analyze the chemical heterogeneity of composite electrospun fibers. The methodology combines spectral segmentation with end member analysis using the N-FINDR algorithm, enabling the identification of components present at concentrations low as 2% and 5%, even in the absence of high- or medium-intensity exclusive Raman bands, respectively. Compared to the traditional approach based on the intensity of exclusive bands, the proposed method demonstrated greater sensitivity for detecting and mapping the distribution of compounds within the fibers.

Spatial segmentation allowed the extraction of average spectra for the components present, which in turn facilitated the identification of weak signals associated with compounds that might otherwise go undetected in the abundance maps.

Raman spectral segmentation allows for the optimization of denoising parameters for each Raman band individually, rather than applying uniform parameters across the entire spectrum. This enables the application of a spectral unmixing algorithm without the need to remove noise from the entire spectrum, by selecting only the bands deemed to contain the most relevant spectral information.

Although the method proposed in this study was validated on electrospun fibers composed of two components, the results may be generalized to fibers containing n components by increasing the number of end members in the N-FINDR algorithm to n + 1.

The main limitations of the method developed in this work include the presence of fluorescent compounds, which can mask Raman bands, and reduced sensitivity in very thin fibers, given that Raman signal intensity is proportional to the number of analyte molecules within the sampling volume [52]. This implies that fibers with smaller diameters generate weaker signals, making their characterization more challenging.

Given that the datasets for end members are constructed to be mutually exclusive, when the principal component is homogeneously distributed within the fibers and minor components are localized in punctual regions, as is the case for PLA and PVA in PLA/PVA fibers, it is challenging to obtain a large number of spectra for the end member dataset. Consequently, weak-intensity bands may not be sufficiently reinforced in the averaged spectra.

Although the proposed method can be applied to polymer blends without exclusive bands, complex polymer blends may lead to potential false positives. Therefore, in the absence of clear spectral evidence, it is recommended to seek complementary evidence supporting the identified component mapping.

Given that, the spatial resolution achievable with confocal Raman microscopy in the lateral directions and along the light path is given by [26]:

$$\Delta x=\Delta y=\frac{0.61\;\lambda }{NA},\;\mathrm{and}\;\Delta z=\frac{0.89\;\lambda }{{\left(NA\right)}^{2}}$$

It would be interesting to explore whether the methodology proposed in this work can be adapted or extended to perform three-dimensional reconstruction of composite electrospun fibers, which could be of interest for analyzing core–shell nanofibrous structures.

Additionally, employing artificial intelligence tools for processing pixels with low intensity and a high spatial signal-to-noise ratio [20,55] would be valuable. These ideas are proposed as future research directions.

Finally, to minimize errors and improve representativeness, it is recommended that measurements should be performed in multiple regions of the material in order to validate the observed chemical heterogeneity.

## Data Availability

The original contributions presented in this study are included in the article. Further inquiries can be directed to the corresponding authors.
